# Joseph Fourier 250th Birthday: Modern Fourier Analysis and Fourier Heat Equation in Information Sciences for the XXIst Century

**DOI:** 10.3390/e21030250

**Published:** 2019-03-06

**Authors:** Frédéric Barbaresco, Jean-Pierre Gazeau

**Affiliations:** 1 Key Technology Domain PCC (Processing, Control & Cognition) Representative, Thales Land & Air Systems, Voie Pierre-Gilles de Gennes, F91470 Limours, France; 2 APC (UMR 7164), Department of Physics, Université Paris-Diderot, F75205 Paris, France

**Keywords:** harmonic analysis on abstract space, heat equation on manifolds and Lie Groups

## Abstract

For the 250th birthday of Joseph Fourier, born in 1768 at Auxerre in France, this MDPI special issue will explore modern topics related to Fourier analysis and Fourier Heat Equation. Fourier analysis, named after Joseph Fourier, addresses classically commutative harmonic analysis. The modern development of Fourier analysis during XXth century has explored the generalization of Fourier and Fourier-Plancherel formula for non-commutative harmonic analysis, applied to locally compact non-Abelian groups. In parallel, the theory of coherent states and wavelets has been generalized over Lie groups (by associating coherent states to group representations that are square integrable over a homogeneous space). The name of Joseph Fourier is also inseparable from the study of mathematics of heat. Modern research on Heat equation explores geometric extension of classical diffusion equation on Riemannian, sub-Riemannian manifolds, and Lie groups. The heat equation for a general volume form that not necessarily coincides with the Riemannian one is useful in sub-Riemannian geometry, where a canonical volume only exists in certain cases. A new geometric theory of heat is emerging by applying geometric mechanics tools extended for statistical mechanics, for example, the Lie groups thermodynamics.

“The differential equations of the propagation of heat express the most general conditions and the physical questions as a result of the analysis of pure problems, which is properly the object of the theory .... The different forms of body are varied to infinity, to the distribution of heat and penetrations; but all the inequalities fade away quickly and disappear as time goes by. The march of the phenomenon become more regular and simpler, is finally subject to a specific law that is the same for all cases, and that it bears no more any sensible imprint of the initial disposition ... The new theories, explained in our work, are united forever with the mathematical sciences, and rest, like them, on invariable foundations; they will retain all the elements they possess today, and they will acquire, continually, more extension”. [Les équations différentielles de la propagation de la chaleur expriment les conditions les plus générales, et ramènent les questions physiques à des problèmes d’analyse pure, ce qui est proprement l’objet de la théorie .... Les formes des corps sont variées à l’infini, la distribution de la chaleur qui les pénètre peut être arbitraire et confuse; mais toutes les inégalités s’effacent rapidement et disparaissent à mesure que le temps s’écoule. La marche du phénomène devenue plus régulière et plus simple, demeure enfin assujettie à une loi déterminée qui est la même pour tous les cas, et qui ne porte plus aucune empreinte sensible de la disposition initiale ... . Les théories nouvelles, expliquées dans notre ouvrage sont réunies pour toujours aux sciences mathématiques et reposent comme elles sur des fondements invariables; elles conserveront tous les éléments qu’elles possèdent aujourd’hui, et elles acquerront, continuellement plus d’étendue.]—Joseph Fourier (1768–1830), Discours préliminaire à la théorie analytique de la chaleur.

[1]

For the 250th birthday of Joseph Fourier (Figure 1) [1,2,3,4,5,6], born in 1768 at Auxerre in France, this MDPI special issue will explore modern topics related to Fourier analysis and Fourier Heat Equation. 

Fourier analysis, named after Joseph Fourier, who showed that representing a function as a sum of trigonometric functions greatly simplifies the study of heat transfer and addresses classically commutative harmonic analysis. Classical commutative harmonic analysis is restricted to functions defined on a topological locally compact and Abelian group G (Fourier series when G = R^n^/Z^n^, Fourier transform when G = R^n^, discrete Fourier transform when G is a finite Abelian group). The modern development of Fourier analysis during XXth century has explored the generalization of Fourier and Fourier-Plancherel formula for non-commutative harmonic analysis, applied to locally compact non-Abelian groups. This has been solved by geometric approaches based on “orbits methods” (Fourier-Plancherel formula for G is given by coadjoint representation of G in dual vector space of its Lie algebra) with many contributors (Dixmier, Kirillov, Bernat, Arnold, Berezin, Kostant, Souriau, Duflo, Guichardet, Torasso, Vergne, Paradan, etc.) [7]. It was observed first by Souriau that the coadjoint orbits carry a natural symplectic structure and there is a closed non-degenerate G-invariant 2-form on each orbit, called the Kirillov-Kostant-Souriau symplectic form that plays a central role in geometric quantization and classification of the homogeneous symplectic manifolds. In parallel, theory of coherent states (Klauder, Perelomov, Gilmore, etc.) and wavelets (Grossmann, Daubechies, Meyer, etc.) has been generalized over Lie groups (by associating coherent states to group representations that are square integrable over a homogeneous space) [8]. One should add the developments, over the last 30 years, of the applications of harmonic analysis to the description of the fascinating world of aperiodic structures in condensed matter physics, e.g., quasicrystals and their diffraction spectra [9]. The notions of model set introduced by Y. Meyer, and of almost periodic functions, have revealed themselves as extremely fruitful in this domain of natural sciences. 

The name of Joseph Fourier is also inseparable from the study of mathematics of heat, but it took almost a century for the most brilliant scientists of the nineteenth century—Fourier, Biot, Poisson, Lamé, and Boussinesq [10]—to unravel complexity appearances of the propagation of heat in solids, to develop efficient physical concepts and related instruments of mathematics, and from confusion that constitutes the reality of the calorific phenomena, to clarify a new knowledge of diffusion equation in elastic and crystal domains. It is from the study of thermal energy that the very notion of diffusion related to the parabolic-type equation is born with Fourier and Biot. Fourier’s first memoir at the Academy of Sciences on this subject dates back to 1807, and completed in 1811 by extensive work that was examined by Malus, Haüy, Laplace, Lagrange, and Legendre. Fourier never adhered to reviewers comments and he reprinted his memoir without taking any account of the critics of these censors. It was in 1822 that the “Analytical Theory of Heat” appeared. The Fourier manuscript must be considered rightly as the foundation of mathematical physics. Modern research on heat equation explores the extension of classical diffusion equation on Riemannian, sub-Riemannian manifolds, and Lie groups (i.e., Hall). The heat equation for a general volume form that not necessarily coincides with the Riemannian one is useful in sub-Riemannian geometry, where a canonical volume only exists in certain cases. Jean-Michel Bismut [11] has introduced the concept of hypoelliptic Laplacian (If X is a Riemannian manifold, the hypoelliptic Laplacian is a family of hypoelliptic operators that interpolates between the ordinary Laplacian and the geodesic flow), with the probabilistic counterpart that is an interpolation between Brownian motion and geodesics. Elliptic heat kernel has infinite propagation speed compared to geodesic flow that has a finite propagation speed. On R^3^, Langevin had introduced the Langevin equation to reconcile Brownian motion and classical mechanics. The hypoelliptic diffusion on the total space of the tangent bundle of a Riemannian manifold is a geometric Langevin process that interpolates between the geometric Brownian motion and the geodesic flow. In parallel with Geometric Mechanics, Jean-Marie Souriau [12] has interpreted the temperature vector of Planck as a space-time vector, obtaining, in this way, a phenomenological model of continuous media that presents some interesting properties: The temperature vector and entropy flux are in duality; the positive entropy production is a consequence of Einstein’s equations; the Onsager reciprocity relations are generalized; and in the case of a fluid in the non-relativistic approximation, the model unifies heat conduction and viscosity (equations of Fourier and Navier). This work has been extended by Claude Vallée [13], by constructing a relativistic model of a dissipative continuum that complies with the laws of both mechanics and thermodynamics.

A last comment concerns the fundamental contribution of Fourier analysis to quantum physics: Quantum mechanics with the notion of representation based on spectral properties of basic observables, like position, momentum, energy, and spin; the quantum field theory saw the first steps that emerged from solutions of Maxwell equations viewed as assemblies of harmonic vibrations (“modes”). 

The content of this special issue highlights papers exploring non-commutative Fourier harmonic analysis, hypoelliptic heat equation, and relativistic heat equation in the context of Information Theory and Geometric Science of Information.

“By scrutinizing the history of these two great thoughts, would we find that the foundation of mathematical thermology by Fourier was less prepared than that of celestial mechanics by Newton?” [En scrutant de près l’histoire de ces deux grandes pensées, trouverait-on que la fondation de la thermologie mathématique par Fourier était moins préparée que celle de la mécanique céleste par Newton].

—Auguste Comte, Cours de philosophie positive, t. II, p. 308, published by Bachelier, 1835.

“ We lack this thermodynamics of shapes, needed according to Thom for a true theory of information ” [Il nous manque cette thermodynamique des formes nécessaire selon Thom à une véritable théorie de l’information].

Edgard Morin, La méthode, la nature de la nature; points, ed. du seuil, 1977.

We will introduce to each paper the following, structuring the special issue in two main sessions:

- Four papers on modern Fourier Heat Theory;
- Five papers on extension of Fourier Harmonic Analysis.

## 1. Modern Fourier Heat Theory

The first paper [14], written by F. Barbaresco, deals with Geometric Theory of Heat based on Jean-Marie Souriau Lie Groups Thermodynamics and its extension to define Maximum Entropy (Gibbs) density with higher order moments constraints. In this Souriau model, Planck temperature is described as an element of Lie algebra for the Lie Group acting on the homogeneous Manifold. Souriau has introduced, through the concept of Souriau, non-equivariant coadjoint action of Lie Group on moment map and Souriau cocycle, an invariant metric that is an extension of classical Fisher metric coming from Information Geometry, called, in the paper, Souriau-Fisher metric and vector-valued extension through poly-symplectic model.

The second paper [15], written by Arjan Van der Schaft and Bernhard Maschke, develops a Thermodynamic model initially proposed by Balian and Valentin for symplectization of contact manifolds, and introduces the global geometric definition of a degenerate Riemannian metric on the homogeneous Lagrangian sub-manifold describing the state properties. In the second part of this paper, authors give a geometric formulation of non-equilibrium thermodynamic processes, and the definition of port-thermodynamic systems and interconnection ports.

The third paper of François Gay-Balmaz and Hiroaki Yoshimura [16] presents new results on the variational formulation of nonequilibrium thermodynamics for discrete or continuum systems, and its extension for irreversible processes. These new models are illustrated in the finite dimensional cases, and on the continuum side.

The fourth paper [17], by Tamás Fülöp, Róbert Kovács, Ádám Lovas, Ágnes Rieth, Tamás Fodor, Mátyás Szücs, Péter Ván, and Gyula Gróf, analyzes the non-Fourier heat conduction phenomenon on room temperature and proposes to use the Guyer-Krumhansl equation to replace classical Fourier’s law for room-temperature phenomena in the modeling of heterogeneous materials. Then, generalized heat conduction equations are introduced where Fourier heat conduction is coupled to elasticity via thermal expansion, resulting in a particular generalized heat equation for the temperature field. The last model is deduced from pseudo-temperature concept underlying heat conduction mechanics behind non-Fourier phenomena.

## 2. Extension of Fourier Harmonic Analysis

In the first paper of the second part [18], Hervé Bergeron and Jean Pierre Gazeau implement the so-called covariant integral quantization for Weyl-Heisenberg and affine group symmetries. Any quantization maps linearly function on a phase space to symmetric operators in a Hilbert space, and covariant integral quantization combines operator-valued measure with the symmetry group of the phase space. Covariant means that the quantization map intertwines classical (geometric operation) and quantum (unitary transformations) symmetries. Integral means that all resources of integral calculus are employed when the procedure is applied to singular functions, or distributions, for which the integral calculus is an essential ingredient. This quantization scheme is first reviewed before its specification to the Weyl-Heisenberg and affine groups, and the fundamental role played by Fourier transform in both cases is emphasized. Generalizations of the Wigner-Weyl transform are considered, and many properties of the Weyl integral quantization, commonly viewed as optimal, are shown to actually be shared by a large family of integral quantizations.

The content of the second paper [19], authored by Maurice de Gosson, lies in the continuation of previous works where it was shown that the equivalence of the Heisenberg and Schrödinger pictures of quantum mechanics requires the use of the Born and Jordan quantization rules. It gives further evidence that the Born–Jordan rule is the correct quantization scheme for quantum mechanics. For this purpose, correct short-time approximations to the action functional, initially due to Makri and Miller, are used, and it is shown that they lead to the desired quantization of the classical Hamiltonian.

In the third paper [20], Remco Duits, Erik J. Bekkers, and Alexey Mashtakov consider the Fokker–Planck PDEs (including diffusions) for stable Lévy processes (including Wiener processes) on the joint space of positions and orientations, which play a major role in mechanics, robotics, image analysis, directional statistics, and the probability theory. The exact analytic designs and solutions are known in the 2D case, where they have been obtained using Fourier transform on SE(2). The authors extend these approaches to 3D using Fourier transform on the Lie group SE(3) of rigid body motions. More precisely, they define the homogeneous space of 3D positions and orientations R^3^⋊S^2^:=SE(3)/({0}×SO(2)) as the quotient in SE(3). In their construction, two group elements are equivalent if they are equal up to a rotation around the reference axis. On this quotient, the authors design a specific Fourier transform and apply it to derive new exact solutions to Fokker–Planck PDEs of α-stable Lévy processes on R^3^⋊S^2^. This reduces classical analysis computations and provides an explicit algebraic spectral decomposition of the solutions. The exact probability kernel for α = 1 (the diffusion kernel) is compared to the kernel for α = 12 (the Poisson kernel). Stochastic differential equations (SDEs), for the Lévy processes on the quotient, are set up and the corresponding Monte-Carlo methods are derived. The exact probability kernels are shown to arise as the limit of the Monte-Carlo approximations.

In the fourth paper [21], authored by Adam Brus, Jiří Hrivnák, and Lenka Motlochová, sixteen types of the discrete multivariate transforms, induced by the multivariate antisymmetric and symmetric sine functions, are explicitly developed. Provided by the discrete transforms, inherent interpolation methods are formulated. The four generated classes of the corresponding orthogonal polynomials generalize the formation of the Chebyshev polynomials of the second and fourth kinds. Continuous orthogonality relations of the polynomials, together with the inherent weight functions, are deduced. Sixteen cubature rules, including the four Gaussian, are produced by the related discrete transforms. For the three-dimensional case, interpolation tests, unitary transform matrices, and recursive algorithms for calculation of the polynomials are presented.

In the fifth paper [22], Enrico Celeghini, Manuel Gadella, and Mariano A. Del Olmo present recent results in harmonic analysis in the real line R and in the half-line R+, which show a closed relation between Hermite and Laguerre functions, respectively, their symmetry groups and Fourier analysis. This can be done in terms of a unified framework based on the use of rigged Hilbert spaces. A relation is established between the universal enveloping algebra of the symmetry groups with the fractional Fourier transform. The results obtained are relevant to quantum mechanics as well as to signal processing as Fourier analysis has a close relation with signal filters. In addition, some new results concerning a discretized Fourier transform on the circle are presented. The authors introduce new functions on the circle constructed with the use of Hermite functions with interesting properties under Fourier transformations.

## Figures and Tables

**Figure 1:**
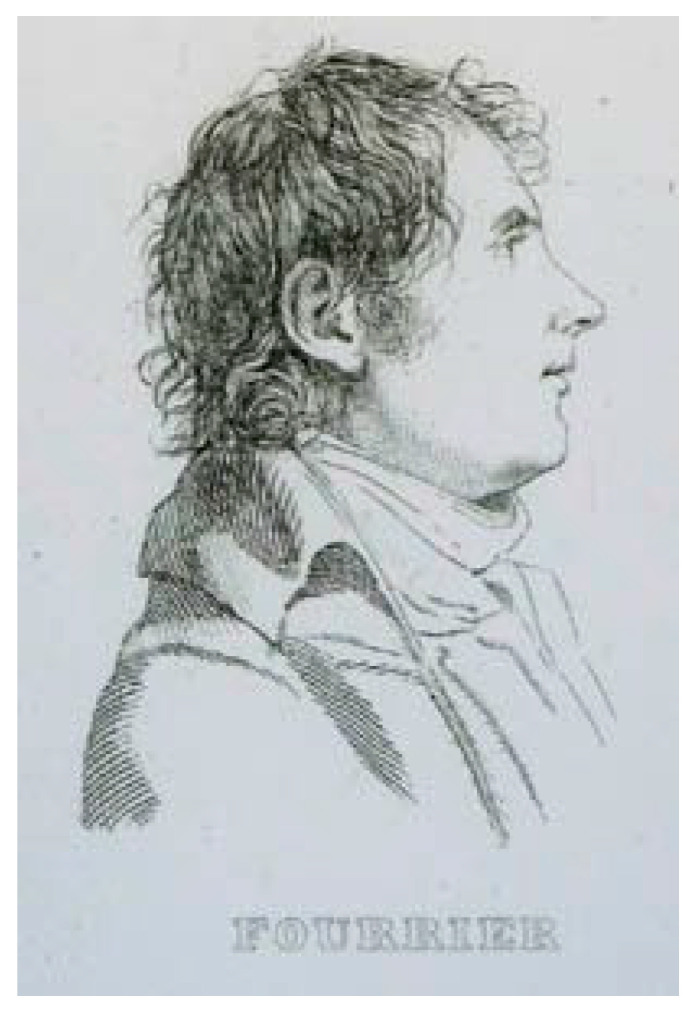
Jean-Baptiste-Joseph Fourier (1768–1830) [1].
